# Targeting TTLL1 Alleviates Aβ-Induced Microtubule Disruption and TAU Pathology in Human iPSC-Derived Cortical Neurons

**DOI:** 10.3390/pharmaceutics18081038

**Published:** 2026-08-20

**Authors:** Mohamed Aghyad Al Kabbani, Laura Köhler, Tamara Wied, Daniel Adam, Jennifer Klimek, Hans Zempel

**Affiliations:** 1 Institute of Human Genetics, Faculty of Medicine and University Hospital Cologne, University of Cologne, 50931 Cologne, Germany; mhd.al-kabbani@uk-koeln.de (M.A.A.K.); laura.koehler@uk-koeln.de (L.K.);; 2 Center for Molecular Medicine Cologne (CMMC), University of Cologne, 50931 Cologne, Germany; 3 Center for Human Genetics and Genomic Medicine, Faculty of Medicine, RWTH Aachen University, 52074 Aachen, Germany

**Keywords:** Alzheimer’s disease, TAU, TTLL, hiPSC neurons, microtubules, neurodegeneration

## Abstract

**Background:** Microtubules play a crucial role in neuronal structure and function, with their stability and dynamics regulated by posttranslational modifications (PTMs) such as polyglutamylation. In Alzheimer’s disease (AD), the microtubule-associated protein TAU becomes mislocalized into the somatodendritic compartment (‘TAU missorting’), dissociates from microtubules, aggregates into neurofibrillary tangles, and contributes to microtubule destabilization and neuronal death. **Objectives and Methods:** Here, we investigated the role of tubulin tyrosine ligase-like proteins (TTLLs) in TAU missorting and microtubule dysregulation using human-induced pluripotent stem cell (hiPSC)-derived cortical neurons treated with oligomeric amyloid-beta (oAβ) to replicate AD-like conditions. *TTLL1*, *TTLL4*, and *TTLL6* were selectively knocked down (KD) to assess their impact on TAU missorting and microtubule stability. Fluorescence resonance energy transfer (FRET) microscopy was used to examine proximities between TAU and TTLL proteins. **Results:** We observed TAU missorting, increased tubulin polyglutamylation, decreased tubulin acetylation associated with microtubule destabilization, and synaptic declustering in oAβ-treated neurons. *TTLL1* KD significantly reduced TAU missorting, tubulin polyglutamylation, and synaptic disintegration, while *TTLL4* KD showed moderate effects, and *TTLL6* KD restored microtubule acetylation. Importantly, *TTLL* KD did not impair neuritic networks, dendritic complexity, or neuronal activity. FRET microscopy in HEK293T cells revealed a close molecular proximity between TAU and TTLL1 consistent with a potential direct or complex-mediated association, but not with other TTLLs, suggesting a direct role of TTLL1 in TAU-mediated toxicity. **Conclusions:** Our findings identify TTLL1 as a promising therapeutic target for limiting TAU-associated cytoskeletal pathology in AD. These results support further development of pharmacological or genetic strategies targeting TTLL1 as a disease-modifying approach for AD and related tauopathies.

## 1. Introduction

Microtubules are cylindrical filamentous heterodimers of α- and β-tubulin that form an essential part of the cytoskeleton. Microtubules play an important role in maintaining neuronal shape and facilitating the transportation of organelles and vesicles across neuronal networks [1]. Microtubule dynamics and functions are tightly regulated by a complex set of posttranslational modifications (PTMs) [2], as well as binding to and interacting with proteins like microtubule-associated proteins (MAPs) and microtubule-severing enzymes such as spastin [3]. In Alzheimer’s disease (AD), microtubules are significantly depleted in affected brains, though the underlying causes remain unclear [4,5].

TAU, a microtubule-associated protein encoded by the *MAPT* gene, is an axon-enriched protein that binds microtubules and promotes their assembly and stability. However, in tauopathies like AD, TAU dissociates from microtubules, missorts to the somatodendritic compartments, and aggregates into hyperphosphorylated neurofibrillary tangles, leading to microtubule fragmentation and neuronal death [6]. Spastin, an ATP-dependent enzyme encoded by the *SPAST* gene, mediates microtubule severing, with its activity regulated by a specific PTM of microtubules called polyglutamylation. This modification involves the addition of glutamate side chains to glutamate residues, usually on the C-terminal tail of tubulin [7]. This modification is carried out by several members of a class of enzymes known as tubulin tyrosine ligase-like proteins (TTLLs), mainly TTLL 1, 4, 5, 6, 7 and 11 [8].

Polyglutamylation is reversible, with cytosolic carboxypeptidases (CCPs) removing glutamate side chains [9]. The balance between adding and removing glutamate chains is essential for health, with hyperglutamylation linked to neurodegeneration. For instance, Purkinje cell degeneration (pcd) mice, in which CCP1 is deficient, exhibit severe neurodegeneration, which is attenuated by knocking out *TTLL1* and *TTLL4*, but not *TTLL5* or *TTLL7* [10,11]. Further evidence for potential involvement of TTLLs in neurodegeneration came from primary rat neurons treated with oligomeric amyloid-beta (oAβ), where missorted TAU appeared to recruit TTLL6 to the somatodendritic compartments, inducing polyglutamylation and spastin-mediated microtubule severing, ultimately resulting in extensive microtubule loss [12,13]. However, the underlying, potentially druggable disease mechanisms by which TAU missorting triggers microtubule dysfunction, as well as the specific TTLLs involved, remain unclear in human disease-relevant models.

Despite decades of research, disease-modifying therapies for AD remain limited. Current therapeutic approaches primarily target amyloid pathology, while interventions directed at downstream cytoskeletal dysfunction and TAU pathology remain underdeveloped. Identifying druggable molecular regulators that preserve neuronal microtubule integrity, therefore, represents an important strategy for developing targeted therapies capable of slowing neurodegeneration. In this study, we first aimed to establish a human tauopathy-relevant model using human-induced pluripotent stem cell (hiPSC)-derived cortical neurons (iNeurons). We treated these neurons with oAβ and observed TAU missorting, increased tubulin polyglutamylation, and synaptic declustering. We then aimed to identify the TTLL(s) responsible for mediating the pathological effects of missorted TAU via individually knocking down *TTLL1*, *TTLL4*, or *TTLL6*. We showed that decreased expression of TTLL1, and to some extent TTLL4, alleviated the pathological effects of oAβ without disturbing the neuritic network, dendritic morphology, or neuronal function. While previous studies have demonstrated that dysregulated tubulin polyglutamylation contributes to TAU pathology [13,14,15], the specific contribution of TTLL1 to this process remains unknown. Here, we provide the first evidence that targeting TTLL1 alone or in combination with another TTLL is a potential therapeutic target in AD and other tauopathies and should be further validated and investigated.

## 2. Methods

### 2.1. hiPSC Maintenance

WTC11 cells with a doxycycline-inducible *neurogenin-2* (*Ngn2*) transgene [16,17] were cultured on Geltrex-coated plates (Thermofisher Scientific, Waltham, MA, USA #A1413302) at 37 °C, 5% CO_2_ and regularly passaged when almost fully confluent using Versene (Thermofisher Scientific, Waltham, MA, USA #15040066) and thiazovivin-supplemented StemMACS iPS-Brew X.F. (Axon Medchem, Groningen, The Netherlands #1535; Miltenyi Biotec, Bergisch Gladbach, Germany #130-104-368) for the first 24 h [4].

### 2.2. Differentiation of hiPSCs into Cortical Neurons (iNeurons)

Differentiation of hiPSCs into cortical neurons was carried out as previously described [17,18,19]. At the start of differentiation, iPSCs were harvested using Accutase (Sigma-Aldrich, St. Louis, MO, USA #A6964-100ML) and seeded onto Geltrex-coated plates in pre-differentiation medium (Thermofisher Scientific, Waltham, MA, USA #12660012) supplemented with thiazovivin (day before differentiation: d − 3). The medium was changed every day for 2 days to fresh pre-differentiation medium without supplementation. On day 0, 50,000 or 300,000 cells were seeded onto poly-D-lysine-coated (Sigma-Aldrich, St. Louis, MO, USA #P7886-50MG) and laminin-coated (Trevigen, Gaithersburg, MD, USA #3446-005-01) 24-well-plates or 6-well plates, respectively, using a maturation medium supplemented with 1:100 GelTrex. Half of the medium was exchanged once per week until analysis.

### 2.3. oAβ Preparation and Treatment

Aβ was prepared and reconstituted into oligomers as described before [13,20]. Briefly, Aβ40 and Aβ42 powder (rPeptide, Watkinsville, GA, USA #A-1153-1 and #A-1163-2, respectively) were completely dissolved in hexafluoro-2-propanol (HFIP) to a final concentration of 1 mM. Following aliquoting, HFIP was evaporated completely using a vacuum concentrator, and the lyophilized powder was stored at −80 °C. On the day of treatment of iNeurons, lyophilized Aβ40 and Aβ42 were redissolved in 50 mM NaOH and mixed to produce an Aβ40/Aβ42 ratio of 7:3 and then diluted with phosphate-buffered saline (PBS) and 50 mM HCl to a final concentration of 100 µM. To induce oligomerization, the Aβ mixture was incubated at 37 °C for one hour. Subsequently, iNeurons on day 21 were treated with 1 µM oAβ or a vehicle control (50 mM HCl and 50 mM NaOH in PBS) for 3 h and analyzed. A new independent batch of oAβ was prepared for each experiment. Figure S1 shows oAβ-treated iNeurons, highlighting the oligomeric state and the distribution and targeting of the oligomers.

### 2.4. Short Hairpin RNA Sequences

For the knockdown of human *TTLL*s in iNeurons, short hairpin RNA (shRNA) oligonucleotides were inserted into pLKO.3G vector (Addgene, Watertown, MA, USA #14748), resulting in a multi-cistronic lentiviral construct expressing green fluorescent protein (GFP) and the corresponding shRNA. The following shRNA sequences were used to target human *TTLL*s or as a control:

Scrambled shRNA (control): 5′-TTGTCTTGCATTCGACTAA-3′

sh*TTLL1*: 5′-GTTTGTGTCTCAATCTAATAA-3′

sh*TTLL4*: 5′-GAGCCTTGGCAATAAGTTC-3′

sh*TTLL6*: 5′-CGGACUCATGAUUUCCAGGATT-3′, 5′-AACAACUCCCUCUUCCAGAAU-3′

### 2.5. Lentiviral-Based Knockdown of TTLLs

Lentivirus particle production and subsequent lentiviral transduction of iNeurons are described in detail in Buchholz et al.’s (2024) study [18]. Briefly, HEK293T cells were co-transfected with the corresponding pLKO.3G plasmid, the packaging plasmid psPAX, and the envelope plasmid pMD2.G (Addgene, Watertown, MA, USA #12259 and #12260, respectively). Four and five days after transfection, the culture supernatant containing lentivirus was collected, filtered and stored at −80 °C. iNeurons were transduced with the lentiviral particles on day 10 and analyzed 11 days after transduction (day 21).

### 2.6. Western Blot Analysis

For Western blot analysis, iNeurons were lysed in RIPA buffer (Sigma Aldrich, St. Louis, MO, USA #R0278), centrifuged at 16,000× *g* for 20 min at 4 °C, diluted in 5× Laemmli buffer, boiled for 10 min at 95 °C, and then separated on 10% sodium dodecyl sulfate (SDS)–polyacrylamide gels. Afterwards, the proteins were transferred to polyvinylidene fluoride (PVDF) membranes overnight at 4 °C and blocked in 5% bovine serum albumin (BSA) (Carl Roth, Karlsruhe, Germany #8076.4) in Tris-buffered saline with 0.1% Tween (TBS-T). Membranes were incubated with the primary antibody overnight at 4 °C, washed three times with TBS-T, and incubated with the corresponding secondary horseradish peroxidase (HRP)-coupled antibody for 1 h at room temperature. After three washing rounds with TBS-T, the immunoreactions were detected by applying the SuperSignal West Pico Chemiluminescent Substrate (Thermofisher Scientific, Waltham, MA, USA #34580) using a ChemiDoc XRS + system (Bio-Rad, Hercules, CA, USA).

### 2.7. Immunofluorescence Labeling of iNeurons

For immunocytochemistry, iNeurons were fixed with 3.7% formaldehyde in PBS containing 4% sucrose at room temperature for 30 min. Afterwards, cells were permeabilized and blocked in 5% BSA and 0.2% Triton X-100 (Carl Roth, Karlsruhe, Germany #3051.2) in PBS for 10 min. After fixation, iNeurons were stained with primary antibodies at 4 °C overnight. The following day, the coverslips were washed three times with PBS and stained with the corresponding secondary antibodies coupled to an AlexaFluor dye for two hours at room temperature. The coverslips were then washed with PBS and stained with NucBlue (Thermofisher Scientific, Waltham, MA, USA #R37605) for 20 min at room temperature, followed by mounting onto glass slides using Aqua-Poly/Mount (Polysciences, Warrington, PA, USA #18606-20). The slides were dried for 24 h at room temperature and then imaged.

### 2.8. Imaging

The immunostained iNeurons were imaged using a wide-field fluorescence microscope (Axioscope 5, Zeiss, Oberkochen, Germany) with the ZenBlue Pro imaging software (V2.5, Zeiss). The images were analyzed using the ImageJ software (Version 2.14.0/1.54f, National Institutes of Health and the Laboratory for Optical and Computational Instrumentation (LOCI), University of Wisconsin, Madison, WI, USA). To measure the levels of TAU or tubulin PTMs in neurons, regions of interest (ROIs) were manually delineated as profiles of the soma or the dendrite where no other somas or processes overlapped, and the mean fluorescence intensity (MFI) of each ROI was measured.

### 2.9. Neuronal Network Analysis

Fields of iNeurons cultures were imaged at 10× magnification after immunostaining for the axon-enriched neurofilament light chain (NF-L) and the somatodendritic marker microtubule-associated protein 2 (MAP2). The area of NF-L- or MAP2-positive neurites was calculated using the ImageJ software and normalized to the number of transduced nuclei in each field, identified via GFP fluorescence.

### 2.10. Sholl Analysis

Sholl analysis [21] was used to investigate the complexity of dendritic arborization in iNeurons immunostained for MAP2. The center of the soma was designated as the center of concentric circles, and the number of intersections was analyzed via the Neuroanatomy plugin in the ImageJ software. Sholl profiles were created by plotting the number of intersections against the distance from the soma (µm). For statistical comparison, either the area under the curve (AUC) or the full-width at half-maximum of the Sholl profiles was compared. The data were tested for normality by the Shapiro–Wilk test and compared using a pairwise *t*-test. Analysis was performed using R (v4.4.1) [22]; AUC was calculated using the DescTools package [23].

### 2.11. Microelectrode Array Measurements

For microelectrode array (MEA) measurements, iPSCs were seeded on MEA 24-well plates and differentiated into cortical neurons as described above. On day 10, iNeurons were transduced with lentiviral particles carrying the corresponding shRNA. Spontaneous activity was recorded for 2 min on day 21 at 37 °C.

### 2.12. Fluorescence Resonance Energy Transfer (FRET) Assay

A live-cell imaging-based FRET assay was carried out to assess molecular proximities between TAU and different TTLLs. HEK293T cells were co-transfected for 24 h with a teal fluorescent protein (TFP)–TAU construct (FRET donor) and yellow fluorescent protein (YFP)–TTLL1, –TTLL4, or –TTLL6 constructs (FRET acceptor). HEK293T cells were also transfected for 24 h with empty vectors expressing TFP or YFP as controls. Live-cell imaging was performed with an inverted Leica DMi8 microscope with the help of Leica LAS X software (v3.7.3). Cells co-expressing both TFP and YFP below saturation levels were selected for analysis. FRET efficiency was analyzed via FRET and the Colocalization Analyzer plugin [24] in the ImageJ software. Briefly, bleed-through (BT) of the donor and acceptor was corrected by quantifying the mean fluorescence intensities (MFI) in cells expressing only the donor or the acceptor, and then calculating the ratio between the MFIs in the FRET channel and the donor of the acceptor channel. FRET efficiency was calculated using the following equation:

FRET efficiency: (MFI_FRET_ − BT_Donor_ × MFI_Donor_ − BT_acceptor_ × MFI_Acceptor_)/√ (MFI_Donor_ × MFI_Acceptor_)

### 2.13. Statistical Analysis

GraphPad Prism (v9.5.1, GraphPad Software, Boston, MA, USA) was used for statistical analysis. The Shapiro–Wilk test was performed to test for a normal distribution of the data. In the case of a normal distribution, statistical analysis was performed by an unpaired *t*-test to compare the means of two groups, or one-way ANOVA with correction for multiple comparisons (Tukey’s test) to compare three or more groups. When the data were not normally distributed, the Mann–Whitney U test or the Kruskal–Wallis test with correction for multiple comparisons (Dunn’s test) were carried out, respectively. Statistical significance was denoted by a significance level of *p* < 0.05. The multiple comparisons between all the different groups across all the experiments included in the study is shown in Figure S4.

### 2.14. Antibodies

The antibodies used in this study are listed in Table 1.

## 3. Results

### 3.1. Establishment of Aβ-Induced TAU Pathology in iNeurons

Previous insights into the potential connection between TAU missorting, microtubule instability, and TTLLs were previously derived from rodent models, whereas a representative human neuronal model was lacking. In order to establish a human tauopathy-relevant model to study TAU-based effects on microtubules, human iPSCs were differentiated into cortical neurons. Briefly, a genetically modified WTC11 iPSC line harboring a doxycycline-inducible *neurogenin-2* (*Ngn2*) transgene was induced to differentiate into pure glutamatergic neuronal cultures (see the Methods Section in this paper; [17,18]).

**Figure 1:**
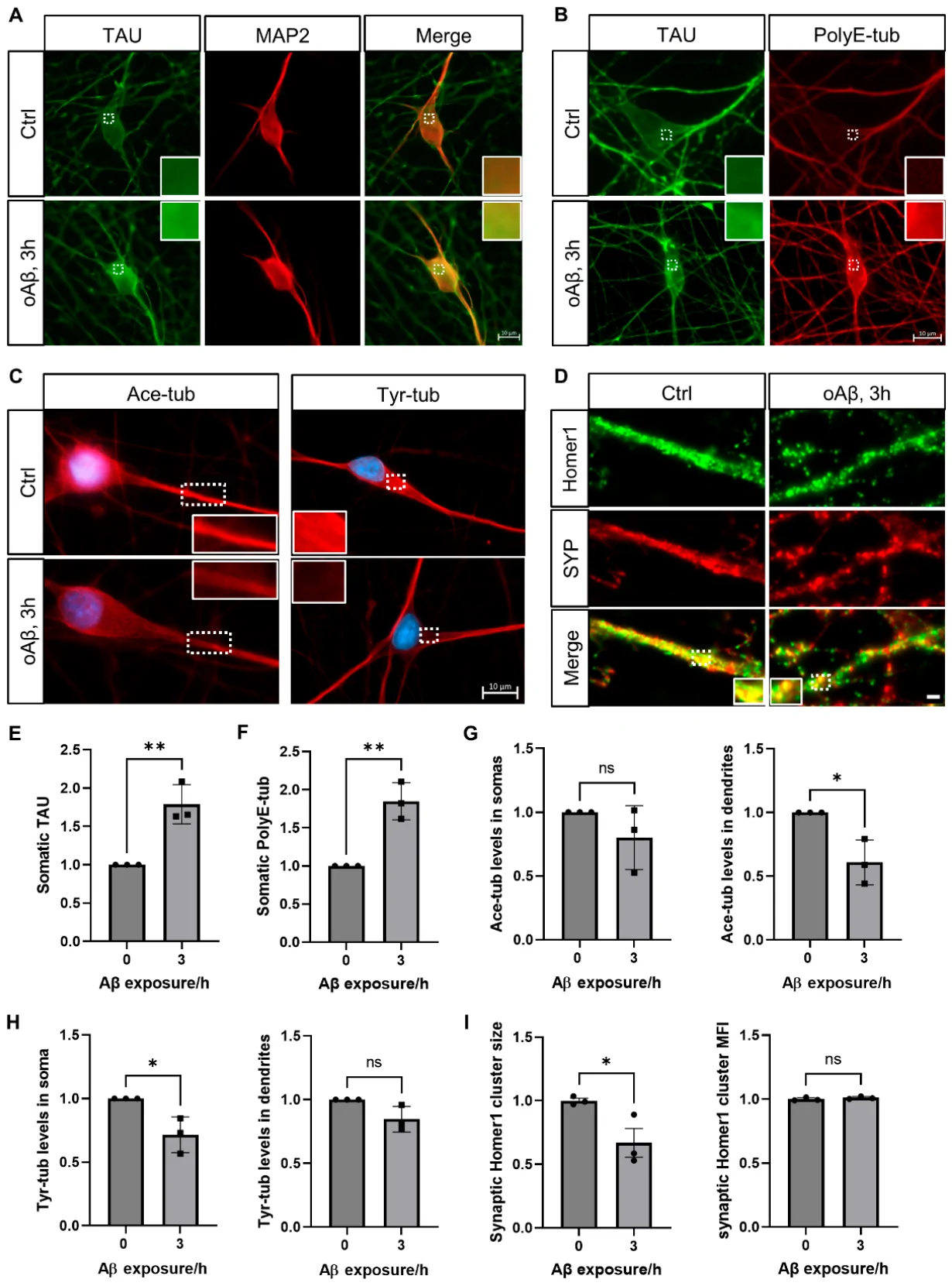
oAβ treatment induces TAU missorting, alters microtubule modifications, and disrupts synaptic clusters in iNeurons. (**A**) Co-immunostaining of TAU and MAP2 in iNeurons treated with oAβ for 3 h or untreated control (Ctrl). Insets display 4-fold magnifications of areas framed with dashed lines, highlighting TAU missorting into the somatic compartment. Scale bar: 10 µm. (**B**) Co-staining of TAU and polyglutamylated tubulin (polyE-tub) in iNeurons treated with oAβ for 3 h or untreated control. Insets show 4-fold magnifications of areas framed with dashed lines, emphasizing increased polyglutamylation in oAβ-treated neurons. Scale bar: 10 µm. (**C**) Immunostaining of acetylated tubulin (ace-tub) and tyrosinated tubulin (tyr-tub) in iNeurons treated with oAβ for 3 h or untreated control. Insets show 3-fold magnifications of areas framed with dashed lines, demonstrating reduced acetylation and tyrosination post-treatment. Scale bar: 10 µm. (**D**) Co-immunostaining of Homer1 and synaptophysin (SYP) in iNeurons treated with oAβ for 3 h or untreated control. Insets show 2-fold magnifications of the areas framed with dashed lines, illustrating the disassembly of synaptic clusters. Scale bar: 2 µm. (**E**) Quantification of somatic TAU levels in (**A**). (*N* = 3 biological replicates comprising three independent iPSC neuronal differentiations; *n* = 15 neurons per biological replicate.) (**F**) Quantification of somatic polyE-tub levels in (**B**). (*N* = 3 biological replicates comprising three independent iPSC neuronal differentiations; *n* = 15 neurons per biological replicate.) (**G**) Quantification of somatic and dendritic ace-tub levels in (**C**). (*N* = 3 biological replicates comprising three independent iPSC neuronal differentiations; *n* = 15 neurons per biological replicate.) (**H**) Quantification of somatic and dendritic tyr-tub levels in (**A**). (*N* = 3 biological replicates comprising three independent iPSC neuronal differentiations; *n* = 15 neurons per biological replicate.) (**I**) Quantification of SYP-colocalizing Homer1 cluster size and mean fluorescence intensity (MFI) in (**D**). (*N* = 3 biological replicates comprising three independent iPSC neuronal differentiations; *n* = 50 puncta per biological replicate.) Shapiro–Wilk test was performed to test for normal distribution of data; statistical analysis was performed by unpaired *t*-test. ^ns^ non-significance, * *p* ≤ 0.05, and ** *p* ≤ 0.01.

Day-21 iNeurons treated with 1 μM of oAβ showed increased levels of somatic TAU, indicative of TAU missorting (Figure 1A,E). This was accompanied by a marked increase in the levels of tubulin polyglutamylation (Figure 1B,F). Interestingly, levels of polyglutamylated tubulin remained unchanged in *MAPT*-knockout iNeurons (characterized in Buchholz et al.’s (2025) study; Figure S2 [25]). To further evaluate microtubule stability in oAβ-treated iNeurons, we investigated the levels of acetylated and tyrosinated tubulin. We observed reduced acetylation in the dendrites and decreased tyrosination in the somatic compartments (Figure 1C,G,H), indicating that microtubule stability and dynamics were further compromised.

We also wanted to test the effects of oAβ on the synaptic integrity of our iNeurons. We, therefore, performed triple staining for Homer1, synaptophysin, and MAP2 to identify dendritic synapses and quantified the size and mean fluorescence intensity of synaptophysin-colocalized Homer1. While the fluorescence intensity of synaptic Homer1 clusters did not change, the size of these clusters was reduced upon oAβ treatment, indicating synaptic destabilization (Figure 1D,I). Hence, oAβ treatment in iNeurons causes TAU missorting, microtubule instability, and synaptic instability, making these neurons a suitable human model for our study.

### 3.2. Reduction in TTLL1 and TTLL4 Expression Attenuates oAβ Toxicity and TAU Missorting

To identify the specific TTLL(s) driving pathological microtubule polyglutamylation upon TAU missorting, we aimed to knock down three polyglutamylating *TTLL*s and subsequently assess the effects of oAβ insult on the levels of polyglutamylated tubulin and the other toxicity readouts established above.

**Figure 2:**
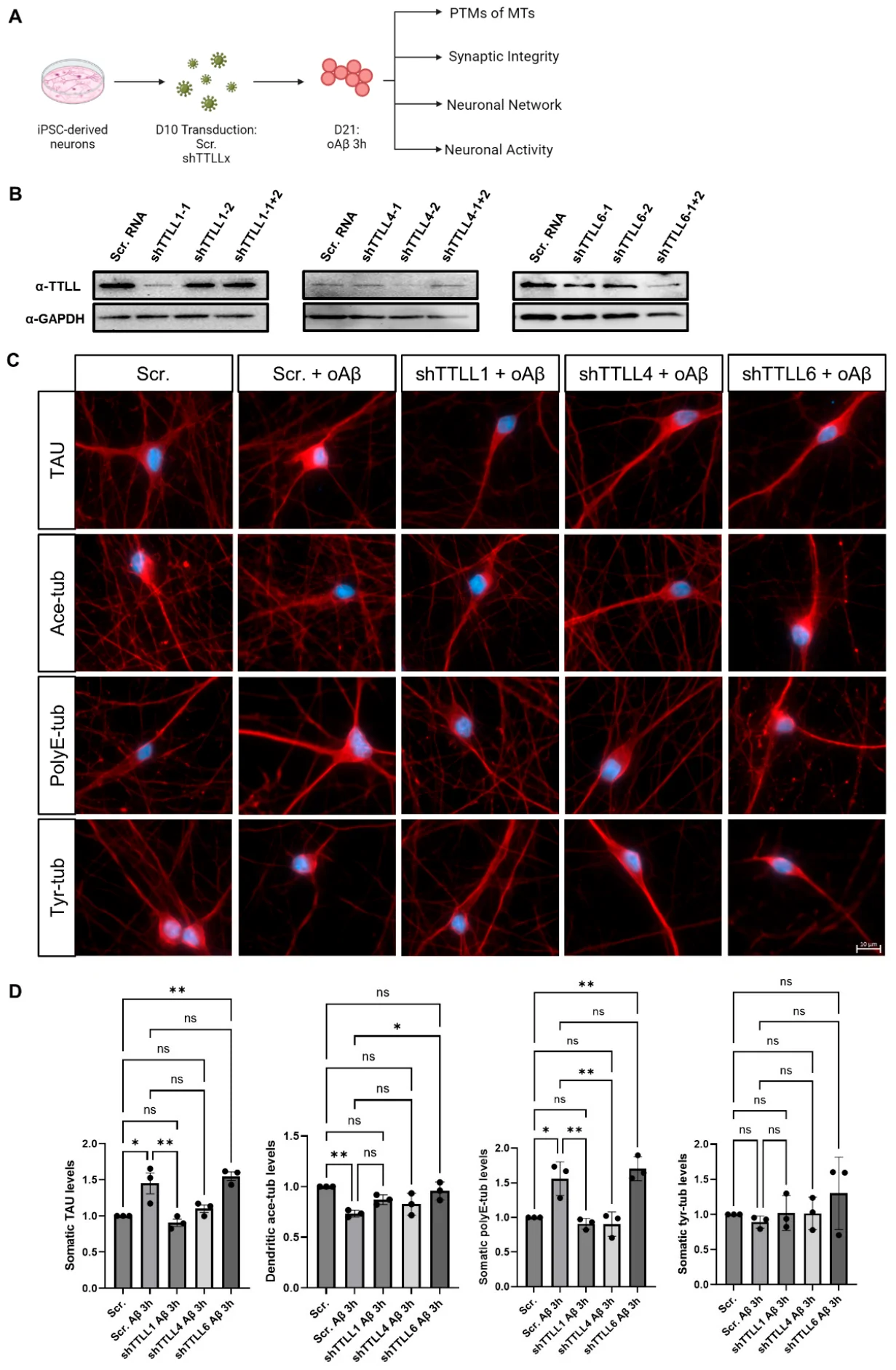
Knockdown of *TTLL1* and *TTLL4* partially ameliorates oAβ-induced pathological changes in iNeurons. (**A**) Schematic representation of the experimental workflow, outlining the knockdown and treatment procedures. Neurons were transduced on Day 10 with shRNA and then treated and fixed as indicated. (**B**) Immunoblotting confirmation of successful knockdown of *TTLL1*, *TTLL4*, and *TTLL6* in iNeurons. (**C**) Immunostaining for TAU, acetylated tubulin (Ace-tub), polyglutamylated tubulin (PolyE-tub), and tyrosinated tubulin (Tyr-tub) in Day-21 iNeurons transduced with scrambled RNA (Scr.) or shRNAs targeting *TTLL1*, *TTLL4*, or *TTLL6* for 11 days, and treated with oAβ or vehicle control for 3 h. Scale bar: 10 µm. (**D**) Quantification of somatic TAU, dendritic ace-tub, somatic polyE-tub, and somatic tyr-tub levels of (**C**). (*N* = 3 biological replicates comprising three independent iPSC neuronal differentiations; *n* = 15 neurons per biological replicate.) Shapiro–Wilk test was performed to test for normal distribution of data; statistical analysis was performed by one-way ANOVA with Tukey’s test for correction of multiple comparisons, except for somatic tyr-tub levels, which were analyzed by Kruskal–Wallis test with Dunn’s test for correction of multiple comparisons. ^ns^ non-significance, * *p* ≤ 0.05, and ** *p* ≤ 0.01.

*TTLL1*, *TTLL4*, and *TTLL6* were individually knocked down using shRNA-lentiviral transduction of Day-10 iNeurons. Effective knockdown was confirmed via Western blotting on Day 21, with residual expression of targeted TTLLs reduced to 20–50% of the control (iNeurons transduced with viruses carrying scrambled shRNA) (Figure 2B and Figure S3).

Following the establishment of efficient lentiviral-based knockdown of *TTLL1*, *TTLL4*, and *TTLL6*, the effects of each of these knockdowns on oAβ-induced toxicity were investigated. Day-10 iNeurons were transduced with the knockdown viruses or a scrambled control. On Day 21, transduced iNeurons were treated with either oAβ or a vehicle control for 3 h, followed by fixation and staining, as above (Figure 2A). Interestingly, oAβ-induced TAU missorting and polyglutamylated tubulin were significantly reduced by *TTLL1* knockdown to near-normal levels, with *TTLL4* knockdown showing a similar effect on polyglutamylation but only a partial effect on TAU missorting. Meanwhile, *TTLL6* knockdown significantly restored acetylated tubulin levels but did not impact TAU missorting or tubulin polyglutamylation. However, the knockdown of none of these *TTLL*s was sufficient to counteract the decrease in tyrosinated tubulin induced by oAβ treatment (Figure 2C,D).

**Figure 3:**
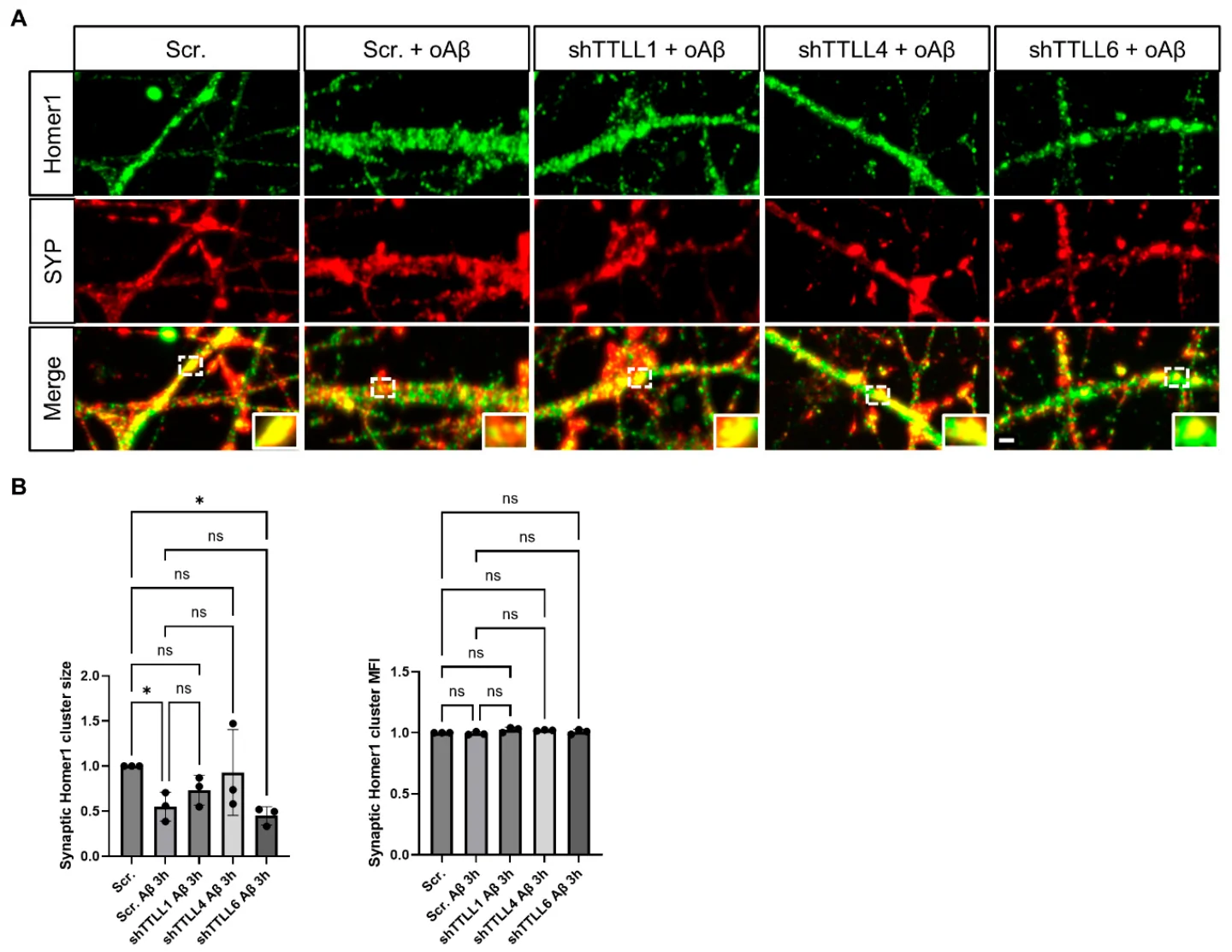
Knockdown of *TTLL1* and *TTLL4* slightly attenuated oAβ-induced synaptic declustering in iNeurons. (**A**) Co-immunostaining of Homer1 and synaptophysin (SYP) in Day-21 iNeurons transduced with scrambled RNA (Scr.) or shRNAs targeting *TTLL1*, *TTLL4*, or *TTLL6* for 11 days and treated with oAβ or vehicle control for 3 h. Insets show 2-fold magnifications of areas framed with dashed lines, highlighting changes in synaptic cluster size. Scale bar: 2 µm. (**B**) Quantification of SYP-colocalizing Homer1 cluster size and mean fluorescence intensity (MFI) in (**A**). (*N* = 3 biological replicates comprising three independent iPSC neuronal differentiations; *n* ≈ 100 puncta per biological replicate.) Shapiro–Wilk test was performed to test for normal distribution of data; statistical analysis was performed by one-way ANOVA with Tukey’s test for correction of multiple comparisons. ^ns^ non-significance; * *p* ≤ 0.05.

While neither *TTLL1* nor *TTLL4* knockdowns fully restored synaptic Homer1 cluster size, they both prevented significant cluster disassembly by 15–30% upon oAβ insult, a protective effect not observed with *TTLL6* knockdown (Figure 3A,B). This indicates that knocking down *TTLL1* and *TTLL4* in iNeurons reduced oAβ-induced TAU missorting, tubulin polyglutamylation, and partially protected synapses, while *TTLL6* KD restored acetylated tubulin but did not affect TAU missorting or synaptic protection, with none preventing tyrosinated tubulin loss.

### 3.3. Knockdown of TTLLs Does Not Impair Neuritic Networks or Neuronal Function

To assess the broader impact of *TTLL* knockdown on neuronal networks, morphology, and function, *TTLL1*, *TTLL4*, or *TTLL6* were knocked down on Day 10, and the effects on neuritic networks and dendritic branching were investigated on Day 21. Axonal and dendritic networks were studied via staining of NF-L and MAP2, respectively.

**Figure 4:**
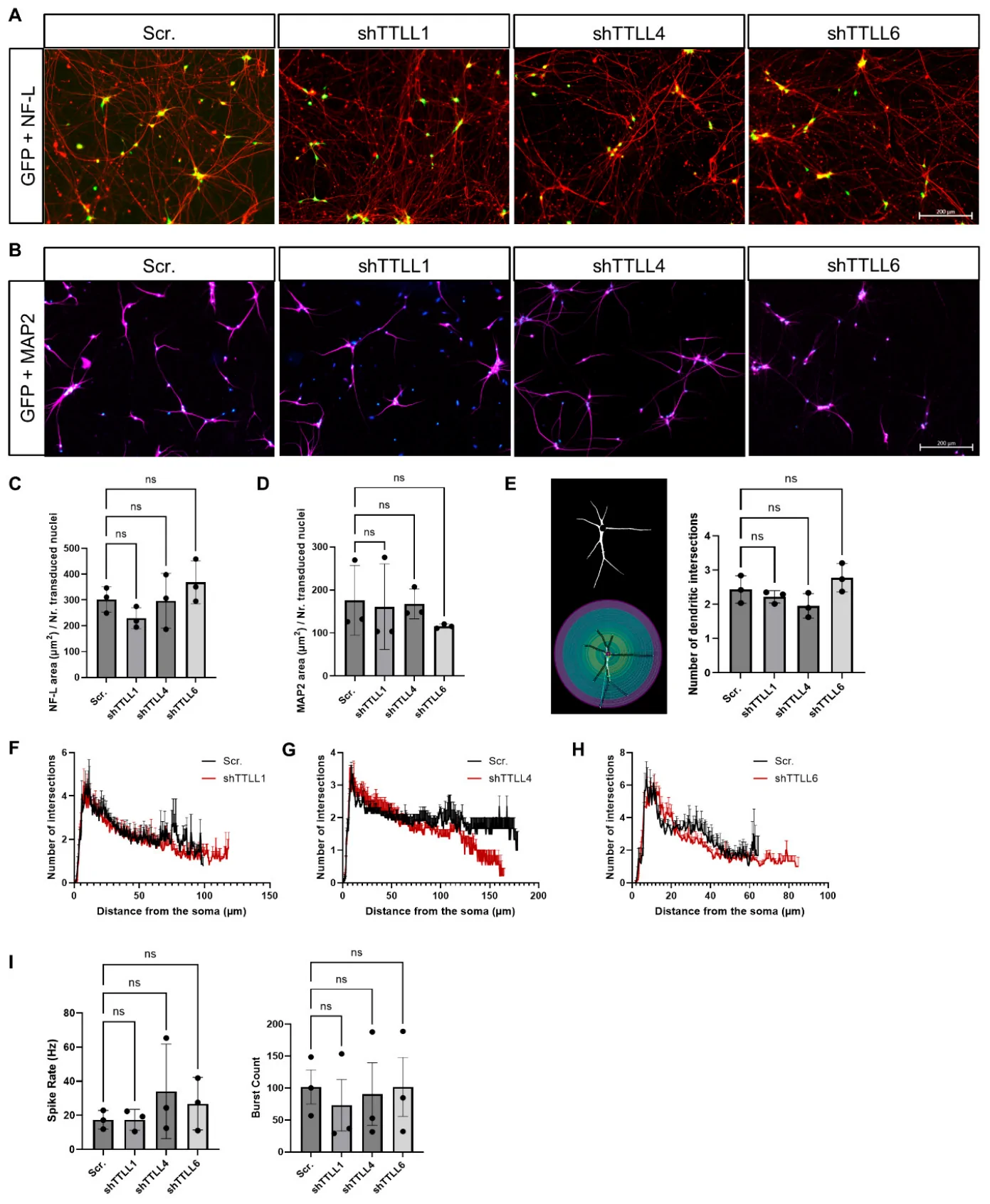
*TTLL* knockdown does not affect neuronal networks, morphology, or function. (**A**,**B**) Immunostaining of neurofilament-L (NF-L) (**A**) or MAP2 (**B**) in Day-21 iNeurons transduced with scrambled RNA (Scr.) or shRNAs targeting *TTLL1*, *TTLL4*, or *TTLL6* for 11 days. Scale bars: 200 µm. (**C**) Quantification of the area covered by the axonal network (NF-L) in (**A**). (*N* = 3 biological replicates comprising three independent iPSC neuronal differentiations; *n* = 5 fields of view at 20× magnification per biological replicate.) ^ns^ non-significance. (**D**) Quantification of the area covered by the dendritic network (MAP2) in (**B**). (*N* = 3 biological replicates comprising three independent iPSC neuronal differentiations; *n* = 5 fields of view at 20× magnification per biological replicate.) ^ns^ non-significance. (**E**) Quantification of the number of dendritic intersections obtained from Sholl analysis. (*N* = 3 biological replicates comprising three independent iPSC neuronal differentiations; *n* = 15 neurons per biological replicate.) ^ns^ non-significance. (**F**–**H**) Sholl profiles showing dendritic branching complexity in Day-21 iNeurons transduced with scrambled RNA (Scr.) or shRNAs targeting *TTLL1* (**F**), *TTLL4* (**G**), or *TTLL6* (**H**) for 11 days. (*N* = 3 biological replicates comprising three independent iPSC neuronal differentiations; *n* = 15 neurons per biological replicate.) (**I**) Quantification of spike rate and burst count from microelectrode array (MEA) recordings in Day-21 iNeurons transduced with scrambled RNA (Scr.) or shRNAs targeting *TTLL1*, *TTLL4*, or *TTLL6* for 11 days. (*N* = 3 biological replicates comprising three independent iPSC neuronal differentiations; *n* = 4–5 wells of a 24-well plate per biological replicate.) Shapiro–Wilk test was performed to test for normal distribution of data; statistical analysis was performed by one-way ANOVA with Tukey’s test for correction of multiple comparisons, except for (**D**), where analysis was performed by Kruskal–Wallis test with Dunn’s test for correction of multiple comparisons. ^ns^ non-significance.

No significant changes were observed in the overall axonal (NF-L staining) or dendritic (MAP2 staining) networks across *TTLL* knockdown conditions compared with the control (Figure 4A–D). Sholl analysis revealed no significant differences in dendritic complexity, although we noted a trend towards longer dendrites in iNeurons with *TTLL1* and *TTLL6* knockdowns (Figure 4E–H). Additionally, neuronal activity, measured by microelectrode array (MEA) recordings, showed no significant alterations in spike rate or burst count across the different knockdown conditions (Figure 4I). This indicates that iNeurons tolerate individual knockdown of the *TTLL*s studied here without obvious impairments in neuronal morphology and function.

### 3.4. TTLL1 and TAU Exhibit Molecular Proximity in HEK293T Cells

To investigate the mechanistic basis behind the ameliorating effects of *TTLL* knockdowns against oAβ-induced toxicity, we applied fluorescence resonance energy transfer (FRET) live-cell imaging to explore molecular proximity and potential interactions between TAU and TTLLs. TFP-TAU and YFP-TTLL1, YFP-TTLL4, or YFP-TTLL6 were co-expressed in HEK293T cells. Co-expression of TFP-TAU and YFP alone, or YFP-TTLL1, YFP-TTLL4, or YFP-TTLL6 and TFP alone, served as negative controls.

**Figure 5:**
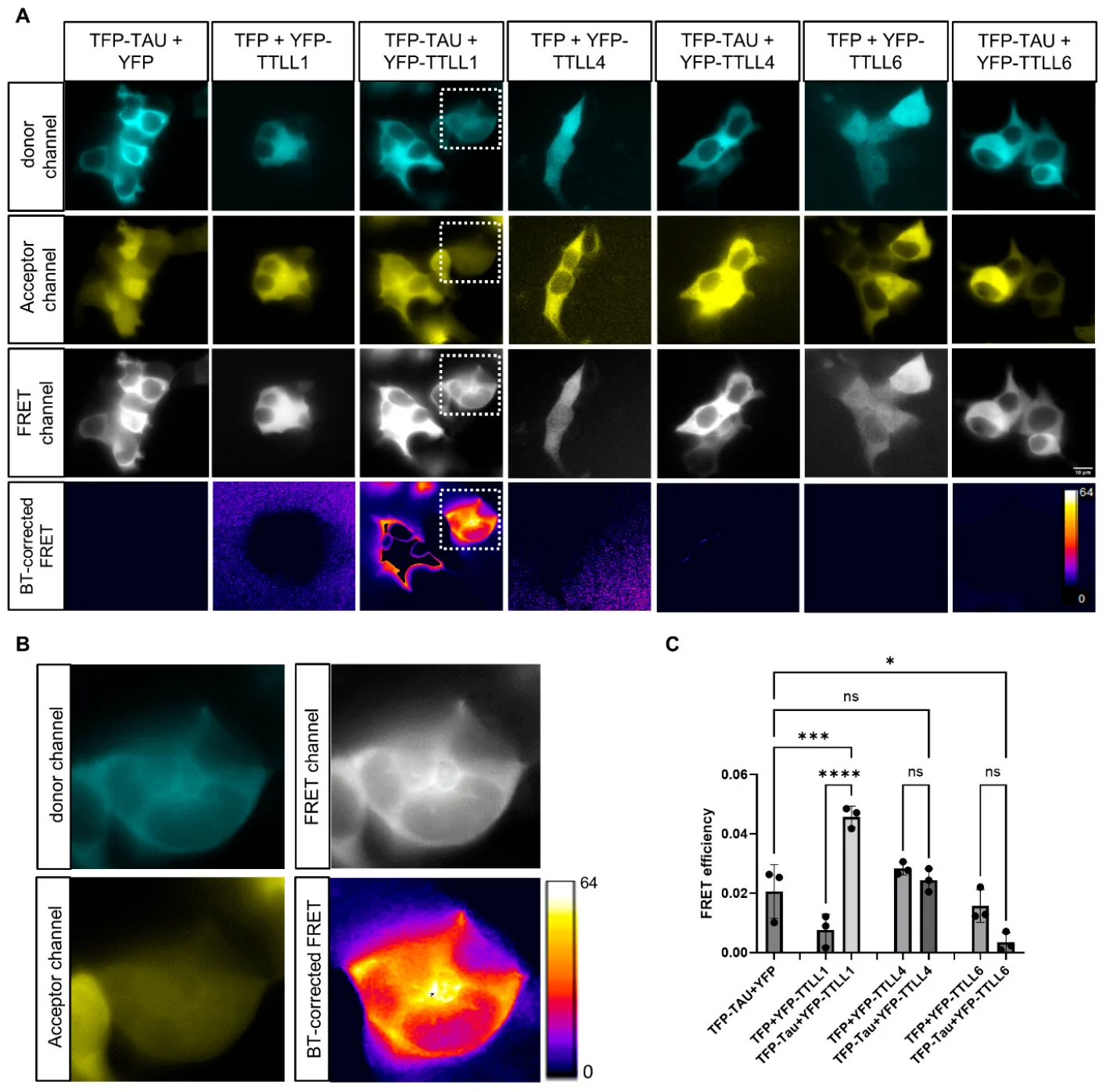
Fluorescence resonance energy transfer (FRET) analysis reveals molecular proximity between TAU and TTLL1. (**A**) FRET microscopy images in HEK293T cells showing various controls (TFP-TAU+YFP, TFP+YFP-TTLL1, TFP+YFP-TTLL4, and TFP+YFP-TTLL6) and experimental conditions (TFP-TAU+YFP-TTLL1, TFP-TAU+YFP-TTLL4, and TFP-TAU+YFP-TTLL6). Images of the three detection channels, donor (TFP), acceptor (YFP), and FRET, as well as the spectral bleed-through (BT)-corrected FRET, are depicted. Scale bar: 10 µm. (**B**) Seven-fold magnification of the area framed by dashed lines in (**A**) showing enhanced FRET signal in low-expressing cells co-transfected with TFP-TAU and YFP-TTLL1. (**C**) Quantification of FRET efficiency from the conditions shown in (**A**). (*N* = 3 biological replicates comprising three independent HEK293T cell cultures; *n* = 5 cells per biological replicate.) Shapiro–Wilk test was performed to test for normal distribution of data; statistical analysis was performed by one-way ANOVA with Tukey’s test for correction of multiple comparisons. ^ns^ non-significance, * *p* ≤ 0.05, *** *p* ≤ 0.001, and **** *p* ≤ 0.0001.

Cells co-transfected with TFP-TAU and YFP-TTLL1 showed significantly higher FRET efficiency compared with the corresponding negative controls, indicating close molecular proximity and suggesting a potential direct or complex-mediated interaction between TAU and TTLL1. In contrast, no significant FRET signal was observed for TTLL4 or TTLL6 compared with their controls (Figure 5). In addition, a preliminary co-immunoprecipitation experiment in iNeurons showed a higher enrichment of TAU in TTLL1 immunoprecipitate compared with several pull-down controls such as GAPDH, synaptophysin, and MAP1B, hinting at a potential direct interaction between TAU and TTLL1 in iNeurons upon oAβ treatment (Figure S6).

## 4. Discussion

This study presents a novel in vitro model of Aβ-induced tauopathy and microtubule impairments using human iPSC-derived cortical neurons. The model effectively recapitulates key features of TAU pathology seen in AD, including TAU missorting, microtubule destabilization, and synaptic defects. By exploring the role of TTLL proteins in this context, we provide new insights into the molecular mechanisms underlying Aβ-induced tauopathy and identify potential therapeutic targets.

oAβ is thought to be the upstream disease-causing agent in AD [26]. Previous studies demonstrated that acute oAβ treatment of rat-derived primary neurons led to TAU missorting, decreased acetylation and increased polyglutamylation of tubulin [13]. The oAβ preparation used in the present study has been extensively characterized in earlier work [13,20] using complementary approaches, including native PAGE, SDS-PAGE, mass spectrometry, electron microscopy, and thioflavin T assays to assess aggregation kinetics. Accordingly, we prepared oAβ following the protocol described in these studies. Furthermore, concentration–response and time-course analyses performed by Kuperstein et al. (2010), Zempel et al. (2013), and Buchholz et al. (2025) [13,20,25] established that treatment with 1 µM of oAβ (at an Aβ40/Aβ42 ratio of 7:3) for 3 h elicits the strongest toxic response while remaining sublethal. Consistent with these previous findings, we observed oAβ-induced TAU missorting to the soma in our human iNeurons, a hallmark of early tauopathy [27]. This pathological shift in TAU localization was accompanied by decreased tubulin acetylation and tyrosination, and increased polyglutamylation. Microtubules are regulated by a complex set of PTMs that govern their dynamics and stability. Decreased acetylation, for instance, has been consistently used as a marker associated with unstable microtubules [28,29], while decreased tyrosination negatively affects neuronal polarity and neurite outgrowth [30]. Polyglutamylation, meanwhile, recruits the microtubule-severing enzyme spastin [7], with hyperglutamylation linked to neurodegeneration and neuronal death [31]. In addition, we observed a significant decrease in the size of synaptophysin-juxtaposing Homer1 clusters following oAβ treatment. Homer1 is a post-synaptic density scaffold protein that is classified as an immediate early gene and is induced by neuronal activity [32]. Synaptic Homer1 cluster disassembly has previously been shown to be driven by Aβ in primary rat neurons, leading to the loss of synaptic structure and function [33]. Taken together, oAβ-insulted iNeurons exhibit detrimental changes in the PTMs of their microtubules, alongside TAU missorting and synaptic declustering, all of which are reminiscent of AD and related tauopathies.

We decided to knock down several glutamylating *TTLL*s in order to pinpoint the one responsible for pathological polyglutamylation and to observe whether abolishing it would mitigate the harmful effects of oAβ. We opted to focus on TTLL1, TTLL4, and TTLL6 because of their expression levels and previously described pathological relevance: TTLL1 is the major polyglutamylating TTLL in the brain [34]; TTLL1 and TTLL4 depletion mitigated neurodegeneration in pcd mice, a model that mainly exhibits adult-onset degeneration of cerebellar Purkinje neurons and selected thalamic neurons [11]; and TTLL6 translocated to dendrites of primary neurons exposed to oAβ, where it mediated polyglutamylation, spastin recruitment, and microtubule loss [13]. In our study, *TTLL1* knockdown significantly protected iNeurons against oAβ-induced TAU missorting, reduced elevated levels of tubulin polyglutamylation, and partially alleviated the dissociation of synaptic clusters, suggesting that *TTLL1* plays a critical role in the early stages of Aβ-induced tauopathy. Meanwhile, overexpression of TTLL1 in iNeurons resulted in elevated levels of somatic TAU reminiscent of TAU missorting (Figure S5), highlighting a potential toxic feedback loop between the two proteins. *TTLL4* knockdown decreased polyglutamylation levels, but its effect on TAU missorting was less pronounced, indicating a rather secondary role for TTLL4. Interestingly, *TTLL6* knockdown did not significantly affect TAU missorting but was the only knockdown that restored microtubule acetylation, hinting at a potential compensatory mechanism that stabilizes microtubules independently of TAU.

Polyglutamylating TTLLs are important enzymes that regulate neuronal microtubule organization, dynamics, and interactions with other proteins [7,35,36]. Therefore, we speculated that knockdown of different *TTLL*s could impact neuronal morphology, networks, or activity, limiting the possibility of targeting TTLLs therapeutically. To investigate this further, we decided to study the dendritic network and dendritic branching by staining for MAP2, a long-established dendritic marker [37], and the axonal network by staining for NF-L, an intermediate filament highly concentrated in axons and a biomarker of neuro-axonal damage [38]. However, neither neuritic networks nor dendritic branching were affected by knockdown of any of the three *TTLL*s investigated in this study. Additionally, the burst count and spike rate of iNeurons measured by MEA recordings were also not impacted by TTLL depletion, indicating maintained neuronal activity and making TTLLs an attractive therapeutic target for reducing TAU pathology while preserving neuronal health.

In order to understand the mechanism through which TTLL1 could mediate the detrimental effects of oAβ insult and TAU missorting, we decided to investigate molecular proximity and potential associations between TFP-TAU and YFP-tagged constructs of the three TTLLs investigated in this study via FRET microscopy in HEK293T cells. It was previously reported that TAU and TTLL6 overexpressed in HEK293T cells may interact directly [13]. In this study, however, only cells expressing TFP-TAU and YFP-TTLL1 showed FRET efficiency values higher than negative controls. FRET can only occur when the distance between the donor (TFP) and the acceptor (YFP) is less than 10 nm, which indicates that TAU and TTLL1 are in close proximity to each other. This may explain why the most effective protection against oAβ was observed in iNeurons with *TTLL1* knockdown. We hypothesize that missorted TAU following oAβ insult transports TTLL1 to the somatodendritic compartments where it polyglutamylates microtubules, culminating in decreased microtubule stability and synaptic loss. Previous studies have linked Aβ-induced TAU missorting to increased tubulin polyglutamylation, microtubule disruption, and synaptic dysfunction, suggesting a mechanistic connection between these pathological events. In particular, Zempel et al. (2013 and 2017) [13,39] reported that Aβ oligomers induce TAU missorting together with TTLL6 redistribution, increased tubulin polyglutamylation, and subsequent recruitment of the microtubule-severing protein spastin, whereas subsequent work established Aβ-induced disruption of TAU sorting in human neurons [25]. However, the available time-course data do not establish a strict sequential order in which TAU missorting necessarily precedes polyglutamylation, and the relationship between these events may be more complex. Our findings extend this model by identifying TTLL1 as an additional modulator of this pathway in human iPSC-derived cortical neurons, as *TTLL1* knockdown reduced both TAU missorting and tubulin polyglutamylation and was associated with preservation of synaptic integrity. Thus, we propose that TTLL1-dependent polyglutamylation contributes to the pathological processes associated with TAU missorting and synaptic disruption, while recognizing that the precise temporal and causal relationships between these events remain to be fully resolved. This postulated TAU-TTLL1 relationship is a working model supported by proximity data, rather than an established molecular pathway. Interestingly, preliminary co-immunoprecipitation experiments in iNeurons hinted at a direct interaction between TAU and TTLL1, but this warrants further investigation.

## 5. Conclusions

We showed that human iPSC-derived neurons subjected to oAβ suffer from TAU missorting, stability-decreasing changes in microtubule PTMs, and dissociation of synaptic clusters. These noxious effects are significantly reduced via *TTLL1* knockdown, without affecting neuronal networks and activity. Rather than establishing a new role for tubulin polyglutamylation in relation to tauopathy, our findings extend the current understanding of the pathological implications of tubulin hyperglutamylation by identifying TTLL1 as a key polyglutamylase contributing to Aβ-induced TAU pathology in human iPSC-derived cortical neurons. Collectively, our findings establish TTLL1 as a promising disease-modifying therapeutic target upstream of microtubule destabilization and synaptic dysfunction. Pharmacological or nucleic acid-based suppression/inhibition of TTLL1 activity, therefore, represents a promising strategy for therapeutic approaches in Alzheimer’s disease and related tauopathies.

## 6. Limitations

This study has some limitations that should be considered when interpreting the findings. While efficient knockdown of *TTLL1*, *TTLL4*, and *TTLL6* was confirmed at the protein level by Western blot analysis, additional validation using independent RNAi sequences, RNAi-resistant rescue constructs, or catalytically inactive TTLL mutants would further strengthen the specificity of the observed effects and more directly establish the contribution of TTLL glutamylase activity to the development of TAU pathology and microtubule loss. Likewise, transcript-level validation of knockdown efficiency and assessment of potential compensatory regulation among other TTLL family members were beyond the scope of the present study but will be important to address in future investigations.

Second, our conclusions are based on a single engineered human iPSC-derived cortical neuron line exposed to an acute oAβ paradigm. Although this model reproduces key aspects of early AD-related TAU pathology, it represents an acute transient insult rather than a recapitulation of a complex chronic condition. Validation in additional human iPSC lines and in vivo models will be important to determine the physiological relevance of our findings.

Third, the present work focuses on acute cytoskeletal and synaptic responses following oAβ exposure. Consequently, the temporal sequence linking TTLL1-mediated tubulin polyglutamylation, TAU missorting, microtubule alterations, and synaptic dysfunction remains to be fully established. Future studies employing time-course experiments and long-term functional analyses will be required to further define this pathogenic cascade and determine whether TTLL1 depletion provides sustained neuroprotection.

Finally, although *TTLL1* knockdown significantly reduced oAβ-induced tubulin polyglutamylation, TAU missorting, and synaptic pathology, not all disease-associated phenotypes were completely rescued. Therefore, our findings identify TTLL1 as an important modulator of Aβ-induced cytoskeletal pathology rather than establishing it as the sole regulator or a validated disease-modifying therapeutic target. In addition, further investigation of the effects of *TTLL1* KD on neuronal safety is warranted.

## Figures and Tables

**Table 1:** List of the primary and secondary antibodies used in this study.

Antibody	Animal Species	Clonality	Cat#	Supplier	RRID	Use and Dilution
Total TAU (K9JA)	Rabbit	Polyclonal	A0024	Agilent, Santa Clara, CA, USA	AB_10013724	ICC (1:1000)
Acetyl-α-tubulin (Lys40) (D20G3)	Rabbit	Monoclonal	5335	Cell Signaling, Danvers, MA, USA	AB_10544694	ICC (1:500)
Anti-tubulin polyglutamylated antibody (Clone B3: reacts specifically with the glutamylated motif at amino acids 445–457 of the C-terminal region of α-tubulin and detects glutamate side chains with 2 or more glutamate residues)	Mouse	Monoclonal	T9822	Sigma-Aldrich, St. Louis, MO, USA	AB_477598	ICC (1:500)
Anti-tyrosinated-α-tubulin antibody	Rat	Monoclonal	MAB1864-I	Sigma-Aldrich, St. Louis, MO, USA	AB_2890657	ICC (1:500)
GAPDH antibody	Mouse	Monoclonal	sc-365062	Santa Cruz Biotechnology, Dallas, TX, USA	AB_10847862	WB (1:1000)
Anti-MAP2 antibody	Chicken	Polyclonal	ab5392	Abcam, Cambridge, UK	AB_2138153	ICC (1:2000)
Anti-NF-L antibody	Rabbit	Polyclonal	12998-1-AP	Proteintech, Rosemont, IL, USA	AB_10597388	ICC (1:500)
Anti-Homer1 antibody	Rabbit	Polyclonal	12433-1-AP	Proteintech, Rosemont, IL, USA	AB_2295573	ICC (1:200)
Anti-synaptophysin antibody	Mouse	Monoclonal	67864-1-Ig	Proteintech, Rosemont, IL, USA	AB_2918622	ICC (1:200)
Anti-TTLL1 antibody	Rabbit	Polyclonal	PA5-27285	Thermofisher Scientific, Waltham, MA, USA	AB_2544761	WB (1:500)
Anti-TTLL4 antibody	Rabbit	Polyclonal	HPA027091	Sigma Aldrich, USA	AB_10601828	WB (1:500)
Anti-TTLL6 antibody	Rabbit	Polyclonal	PA5-100050	Thermofisher Scientific, Waltham, MA, USA	AB_2815580	WB (1:500)
Anti-chicken secondary antibody, DyLight™ 350	Goat	Polyclonal	SA5-10069	Thermofisher Scientific, Waltham, MA, USA	AB_2556649	ICC (1:1000)
Anti-rabbit secondary antibody, Alexa Fluor™ 488	Donkey	Polyclonal	A-21206	Thermofisher Scientific, Waltham, MA, USA	AB_2535792	ICC (1:1000)
Anti-mouse secondary antibody, Alexa Fluor™ 568	Goat	Polyclonal	A-11031	Thermofisher Scientific, Waltham, MA, USA	AB_144696	ICC (1:1000)
Anti-rabbit secondary antibody, Alexa Fluor™ 568	Donkey	Polyclonal	A10042	Thermofisher Scientific, Waltham, MA, USA	AB_2534017	ICC (1:1000) IHC (1:400)
Anti-rat secondary antibody, Alexa Fluor™ 568	Goat	Polyclonal	A-11077	Thermofisher Scientific, Waltham, MA, USA	AB_2534121	ICC (1:1000)
Anti-chicken secondary antibody, Alexa Fluor™ 647	Goat	Polyclonal	A21449	Thermofisher Scientific, Waltham, MA, USA	AB_2535866	ICC (1:1000)
Anti-mouse secondary antibody, Alexa Fluor™ 647	Donkey	Polyclonal	A-31571	Thermofisher Scientific, Waltham, MA, USA	AB_162542	ICC (1:1000)
Anti-mouse secondary antibody, HRP	Goat	Polyclonal	115-035-003	Jackson ImmunoResearch Labs, West Grove, PA, USA	AB_10015289	WB (1:1000)
Anti-rabbit secondary antibody, HRP	Goat	Polyclonal	7074	Cell Signaling, Danvers, MA, USA	AB_2099233	WB (1:1000)

## Data Availability

The datasets used and/or analyzed during the current study are available from the corresponding author upon reasonable request.

## Supplementary Materials

The following supporting information can be downloaded at https://www.mdpi.com/article/10.3390/pharmaceutics18081038/s1: Figure S1: oAβ localizes to neurites of iNeurons after 3 h of treatment; Figure S2: *MAPT* knockout protects iNeurons against oAβ-induced elevation in tubulin polyglutamylation. Figure S3: Quantification of *TTLL1*, *TTLL4*, and *TTLL6* knockdown efficiency. Figure S4: Multiple comparisons between all the different groups across all the experiments included in the study. Figure S5: TTLL1 overexpression increases somatic TAU levels in iNeurons. Figure S6: Co-IP reveals a potential direct interaction between TTLL1 and TAU in iNeurons.

## Abbreviations

AD	Alzheimer’s disease
AUC	Area under the curve
BSA	Bovine serum albumin
CCP	Cytosolic carboxypeptidase
FRET	Fluorescence resonance energy transfer
GFP	Green fluorescent protein
HFIP	Hexafluoro-2-propanol
HRP	Horseradish peroxidase
hiPSCs	Human-induced pluripotent stem cells
iNeurons	hiPSC-derived neurons
KD	Knockdown
MAP2	Microtubule-associated protein 2
MEA	Microelectrode array
MFI	Mean fluorescence intensity
NF-L	Neurofilament light chain
Ngn2	Neurogenin-2
oAβ	Oligomeric amyloid beta
PBS	Phosphate-buffered saline
Pcd	Purkinje cell degeneration
PTM	Post-translational modification
PVDF	Polyvinylidene fluoride
ROI	Region of interest
SDS	Sodium dodecyl sulfate
shRNA	Short hairpin RNA
TBS-T	Tris-buffered saline with 0.1% Tween
TFP	Teal fluorescent protein
TTLL	Tubulin tyrosine ligase-like
YFP	Yellow fluorescent protein
