# Supplementary material for: Targeting TTLL1 Alleviates Aβ-Induced Microtubule Disruption and TAU Pathology in Human iPSC-Derived Cortical Neurons
Source: Pharmaceutics. 2026 Aug 20;18(8):1038. doi: 10.3390/pharmaceutics18081038 (PMC13516559; doi:10.3390/pharmaceutics18081038)
Supplement: Supplementary file 1 [file pharmaceutics-18-01038-s001.zip › pharmaceutics-4468277-supplementary.pdf]

Knockdown of TTLL1 reduces Aβ-induced TAU pathology in human iPSC-derived cortical neurons

Mohamed Aghyad Al Kabbani[1, 2], Laura Köhler[1, 2], Tamara Wied[3], Daniel Adam[1, 2], Jennifer Klimek[1, 2], Hans Zempel[1, 2, *]

1 Institute of Human Genetics, Faculty of Medicine and University Hospital Cologne, University of Cologne, Cologne, Germany

2 Center for Molecular Medicine Cologne (CMMC), Faculty of Medicine and University Hospital Cologne, University of Cologne, Cologne, Germany

3 Current affiliation: Max Planck Institute for the Biology of Ageing, Cologne, Germany

* Correspondence to: Dr. Dr. Hans Zempel, hans.zempel@uk-koeln.de

Supplemental Materials

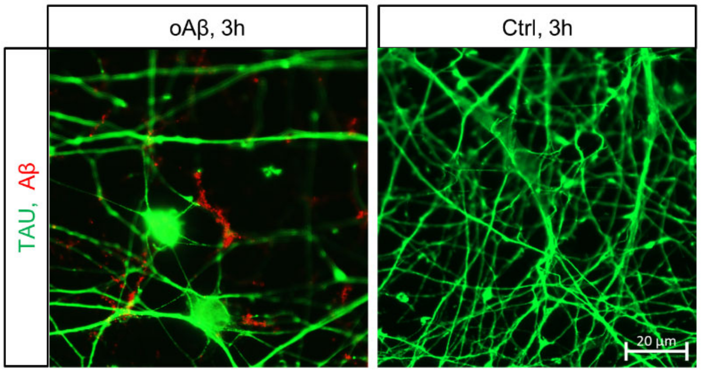

Figure S1. oAβ localizes to neurites of iNeurons after 3 hours of treatment. Immunostaining for TAU and Aβ in oAβ-treated iNeurons, or untreated controls (ctrl). Scale bar: 20 µm.

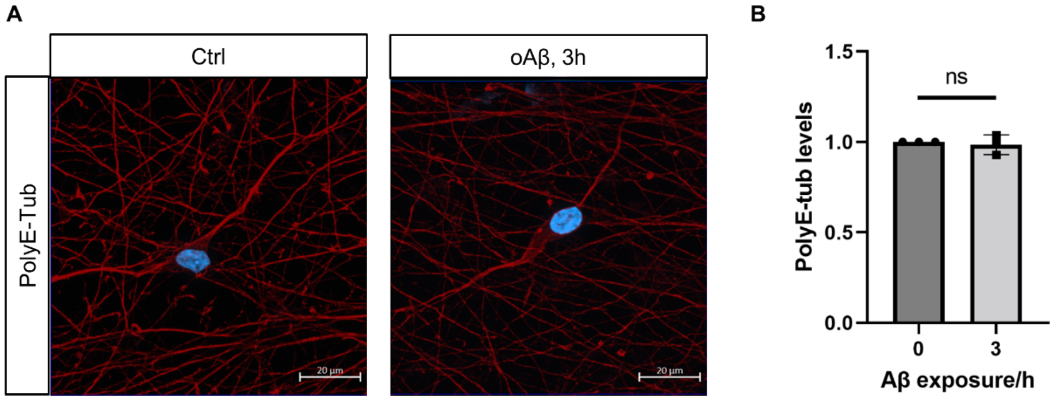

Figure S2. ***MAPT*** **knockout protects iNeurons against oAβ-induced elevation in tubulin polyglutamylation. A)** Immunostaining for polyglutamylated tubulin (PolyE-tub) in *MAPT* KO iNeurons treated with oAβ or untreated control (Ctrl). Scale bar: 20 µm. **B)** Quantification of somatic polyE-tub levels in (A). (N=3 biological replicates comprising three independent iPSC neuronal differentiations, n=15 neurons per biological replicate). Shapiro–Wilk test was performed to test for normal distribution of data; statistical analysis was performed by unpaired *t* test. ^{ns} non-significance

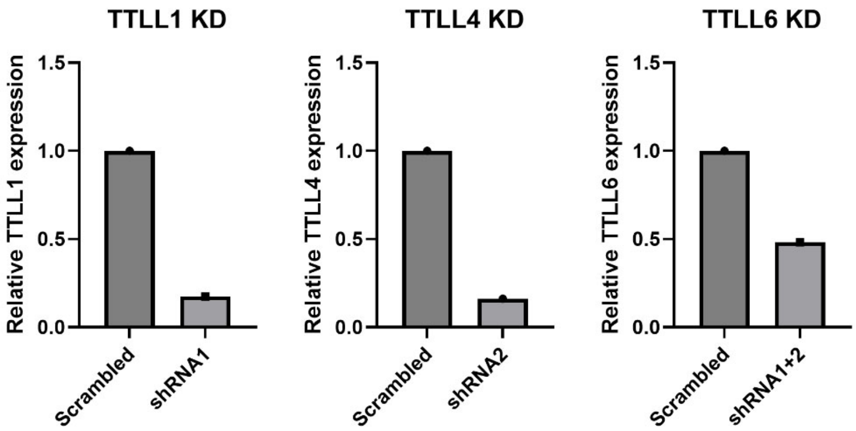

Figure S3. Quantification of TTLL1, TTLL4, and TTLL6 knockdown efficiency. Representative Western blots shown in Figure 2 were quantified by densitometry. TTLL1, TTLL4, and TTLL6 protein levels were normalized to GAPDH loading control.

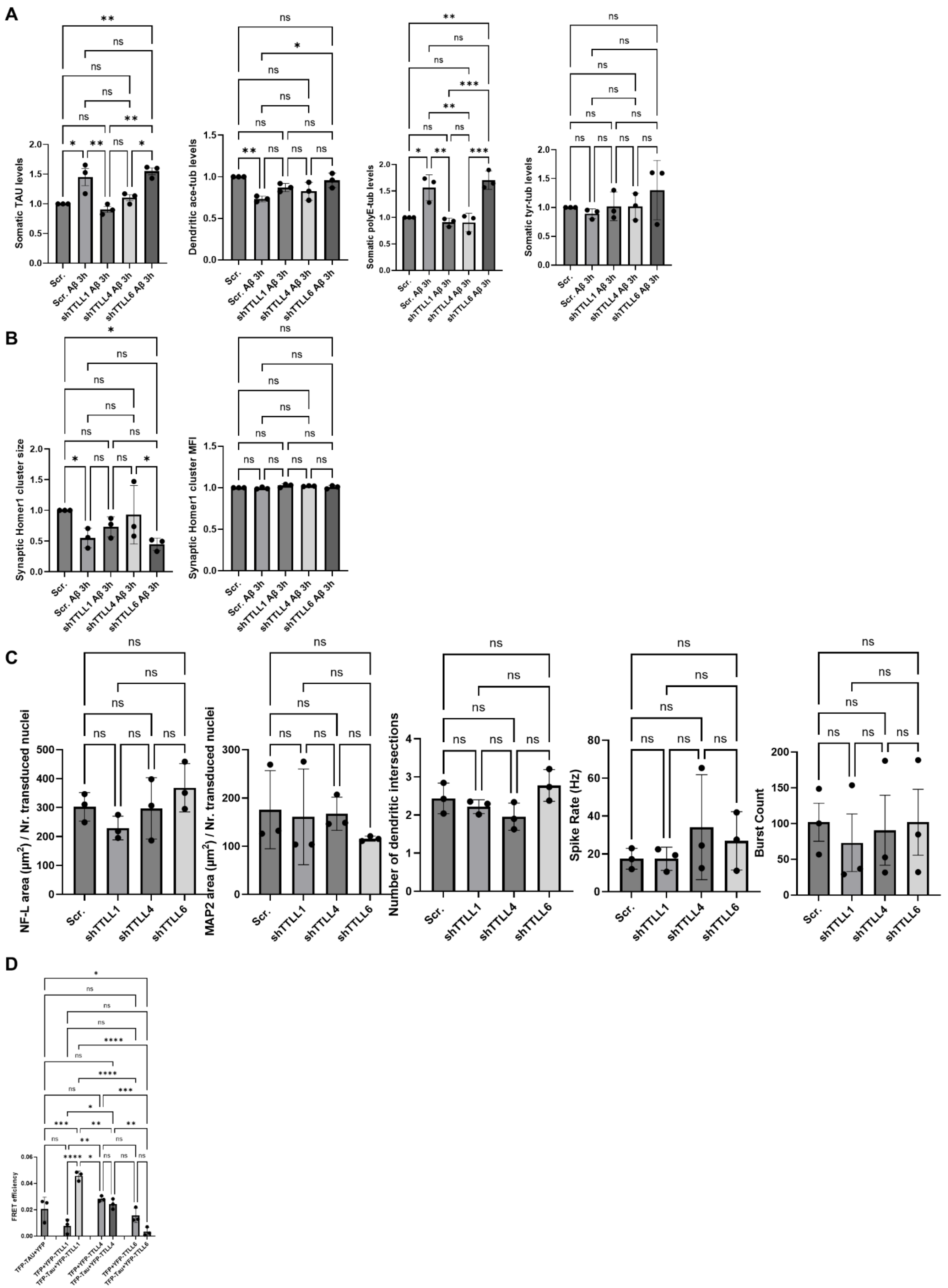
A
Somatic TAU levels
Dendritic ace-tub levels
Somatic polyE-tub levels
Somatic tyr-tub levels
Scr.
Scr. Aβ 3h
shTTLL1 Aβ 3h
shTTLL4 Aβ 3h
shTTLL6 Aβ 3h
B
Synaptic Homer1 cluster size
Synaptic Homer1 cluster MFI
C
NF-L area (µm²) / Nr. transduced nuclei
MAP2 area (µm²) / Nr. transduced nuclei
Number of dendritic intersections
Spike Rate (Hz)
Burst Count
shTTLL1
shTTLL4
shTTLL6
D
FRET efficiency
TFP-TAU+YFP
TFP+YFP-TTLL1
TFP-Tau+YFP-TTLL1
TFP+YFP-TTLL4
TFP-Tau+YFP-TTLL4
TFP+YFP-TTLL6
TFP-Tau+YFP-TTLL6

Figure S4. Multiple comparisons between all the different groups across all the experiments included in the study. Shapiro-Wilk test was performed to test for normal distribution of the data. In case of normal distribution, statistical analysis was performed by one-way ANOVA with correction for multiple comparisons (Tukey's test) to compare three or more groups. When the data were not normally distributed, Kruskal-Wallis test with correction for multiple comparisons (Dunn's test) were carried out. ^{ns} non-significance, * P ≤ 0.05, ** P ≤ 0.01, *** P ≤ 0.001, **** P ≤ 0.0001.

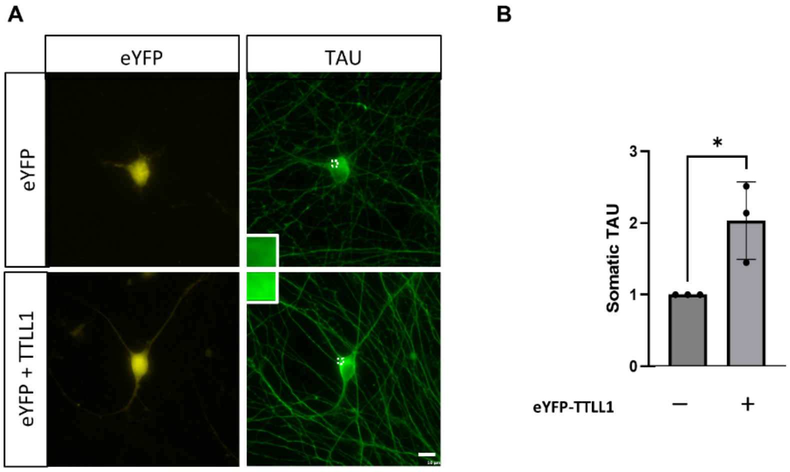

Figure S5. TTLL1 overexpression increases somatic TAU levels in iNeurons. A) Fluorescent eYFP signal and TAU immunostaining in Day 21 iNeurons transduced with either a transduction control or eYFP-tagged TTLL1 for 16 days. eYFP marks transduced neurons. Insets show magnification of areas framed with dashed lines, highlighting increased somatic TAU localization upon TTLL1 overexpression. Scale bar: 10 µm. **B)** Quantification of somatic TAU levels in (A). (N=3 biological replicates comprising three independent iPSC neuronal differentiations, n=15 neurons per biological replicate). Shapiro–Wilk test was performed to test for normal distribution of data; statistical analysis was performed by unpaired *t* test. *P ≤ 0.05.

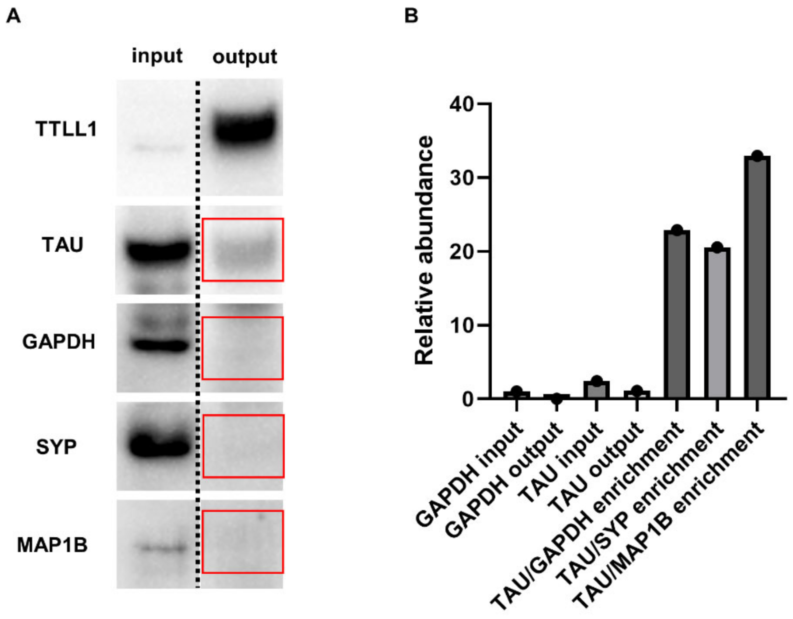

Fig S6. Co-IP reveals a potential direct interaction between TTLL1 and TAU in iNeurons. A) Western blot showing immunoprecipitation of TTLL1 from neuronal lysates and detection of co-immunoprecipitated TAU. Input samples represent total cell lysates prior to immunoprecipitation. GAPDH, synaptophysin (SYP), and MAP1B were assessed as negative controls for nonspecific protein pull-down. **B)** Quantification of TAU enrichment in the TTLL1 immunoprecipitate relative to input. Band intensities were quantified by densitometry and normalized to the corresponding input signal, followed by a second normalization to the enrichment levels of GAPDH, SYP, or MAP1B.
